# In vivo model of human post-traumatic heterotopic ossification demonstrates early fibroproliferative signature

**DOI:** 10.1186/s12967-019-1996-y

**Published:** 2019-08-02

**Authors:** Jaira F. de Vasconcellos, Sonia Zicari, Stephen D. Fernicola, Daniel W. Griffin, Youngmi Ji, Emily H. Shin, Patrick Jones, Gregory T. Christopherson, Husain Bharmal, Carl Cirino, Thao Nguyen, Astor Robertson, Vincent D. Pellegrini, Leon J. Nesti

**Affiliations:** 1 Department of Surgery, Walter Reed National Military Medical Center & Uniformed Services University of Health Sciences, 4801 Rockville Pike, Bethesda, MD 20889 USA; 2 Henry M. Jackson Foundation for the Advancement of Military Medicine, 6720A Rockledge Drive, Bethesda, MD 20817 USA; 3 Department of Orthopaedic Surgery, University of Maryland Medical Center, 22 S. Green St., Baltimore, MD 21201 USA; 4 Department of Orthopaedic Surgery, Medical University of South Carolina, 171 Ashley Ave, Charleston, SC 29425 USA; 5 Department of Surgery, Uniformed Services University of the Health Sciences, 4301 Jones Bridge Road, Room A3008C, Bethesda, MD 20892-8022 USA

**Keywords:** Heterotopic ossification, HO, Rat blast model, Fibrosis

## Abstract

**Background:**

The relationship between the tissue injury healing response and development of heterotopic ossification (HO) is poorly understood. Here we compare a rat blast model and human traumatized muscle from a blast injury to study the early signatures of osteogenesis and fibrosis during the formation of HO.

**Methods:**

Rat and human tissues were characterized using histology, scanning electron microscopy, immunohistochemistry, as well as gene and protein expression analysis. Additionally, animals and humans were assessed radiographically for HO formation following injury.

**Results:**

Markers of bone formation were dramatically increased in tissue samples from both humans and rats, and both displayed increased fibroproliferative regions within the injured tissues and elevated expression of markers of tissue fibrosis such as TGF-β1, Fibronectin, SMAD3 and PAI-1. Markers of inflammation and fibrosis (*ACTA*, *TNFα*, *BMP1* and *BMP3*) were elevated at the RNA level in both rat and human samples. By day 42, bone formation in the rat blast model appeared similar in radiographs compared to human patients who progressed to develop post-traumatic HO.

**Conclusions:**

Our data demonstrates that a similar early fibrotic response is evident in both the rat blast model and the human tissues following a traumatic injury and demonstrates the relevance of this animal model for future translational studies.

## Background

Human musculoskeletal injury is commonly associated with trauma and is followed by either a robust healing response involving tissue regeneration or fibrosis [1–4]. In many instances, for example, this healing program results in the creation of functional tissue (e.g. muscle and/or bone regeneration) or can result in the formation of scar tissue [1–4]. In some situations, following muscle trauma, such as that observed in recent wartime wounds, the healing response may lead to aberrant bone formation in the soft tissues, which is termed heterotopic ossification (HO) [5–7]. The incidence of ectopic bone formation following musculoskeletal injury (post-traumatic HO) is a relatively infrequent event but it becomes more common with higher energy injuries and with concomitant central nervous system trauma [8, 9]. Post-traumatic HO is now known to be a common event following musculoskeletal injury sustained in modern combat [10–13]. There is a documented incidence of approximately 60% among high-energy blast-injured patients wounded during the recent war efforts in Iraq and Afghanistan [10–13]. Post-traumatic HO is a significant complication and it is often associated with pain, skin breakdown, impaired prosthetic fitting, decreased joint range-of-motion, neurovascular compromise and ultimately a dysfunctional outcome, culminating in a poor quality of life [7, 12, 14, 15].

Currently, the molecular basis leading to HO remains unclear and there are no universally acceptable interventions for disease prevention. Further, it is not possible to correctly predict which patients surviving blast-injuries will develop HO; thus, there is an unmet need to better understand the molecular mechanisms leading to the onset of HO, to develop predictive biomarkers, novel treatment options and preventive strategies. To more thoroughly understand the process by which HO forms, we have utilized a previously described and validated rat blast injury model that appears to reproduce the pathologic factors necessary for the development of post-traumatic HO in the blast-injured limb [16–18]. In this model, Sprague–Dawley rats develop HO in the residual limb following extremity blast amputation without addition of any exogenous osteogenic agents. The development of disease in the rat model closely mimics the human pathology of HO [16]. The goal of this study was to demonstrate similarities in the early cellular and molecular markers that may lead to HO formation between the in vivo HO rat blast model and human traumatized muscle from patients following blast injury who progressed to develop HO. In particular, we have focused on the role of the fibrotic response as a prelude to bone formation in both humans and the rat model, given the strong link between fibrosis and bone development in HO [19–23]. The results presented here should provide substantial justification for using this rat blast model to gain a better understanding of HO pathogenesis and lead to the identification of predictive biomarkers for post-traumatic HO and novel therapeutic strategies.

## Methods

### Rat blast model and tissue collection

Using a previously described hind limb rat protocol, Sprague–Dawley rats (n = 8) were first anesthetized with ketamine (80 mg/kg) and xylazine (7 mg/kg) delivered intraperitoneally, and pre-blast doses of enrofloxacin (5 mg/kg) and buprenorphine (0.05 mg/kg) were administered subcutaneously for antisepsis and analgesia, respectively, as previously described [16]. Animals were then secured to an aluminum block with a 2.5 cm hole. The limb to be traumatically amputated was held in place over this hole [16]. Immediately afterwards, these injured rats were transferred to sterile field for wound management and primary surgical closure. A detailed description of the blast setup, blast, surgical closure and post blast care has been previously reported [16]. Four rats were sacrificed at 7 days post-injury and the tissue was collected for analysis. Under sterile conditions, muscle samples (~ 0.2 cm^3^) from the zone of the injury of the traumatized residual limb of the rats were surgically removed and analyzed as detailed below. Control uninjured muscle tissue RNA was harvested from uninjured lower extremities. In the acute post-operative period, rats were monitored for signs of distress, and given a 5-day course of buprenorphine (0.05 mg/kg administered subcutaneously twice a day) and enrofloxacin (5 mg/kg administered subcutaneously twice a day). All remaining animals (n = 4) were euthanized 42 days after the blast injury, to be used for radiographic evaluation of the injury site. Radiographs of the amputated limbs were performed with a digital Faxitron radiography machine (Faxitron X-Ray LLC, Lincolnshire, IL) as previously described [16]. All animal procedures were performed under approved appropriate protocols by the Institutional Animal Care and Use Committee at the University of Maryland Medical Center.

### Human tissue collection and radiographs

Discarded muscle tissues (~ 0.2 cm^3^ in volume) were obtained from the zone of injury of extremities from 5 wounded patients during surgical debridement procedures within 2-weeks of blast trauma (average 10-days post-injury). Tissue samples were divided into equal portions for histology and RNA extraction. This collection method was performed with patient consent according to a protocol approved by the Institutional Review Board at Walter Reed National Military Medical Center. Human control uninjured muscle tissue RNA was purchased from Amsbio (Cambridge, MA). Human radiographs of the injured extremity were performed following standard clinical protocols at the Walter Reed National Military Medical Center as part of the patient’s clinical care, including in preparation for the debridement procedure (average 10-days post-injury) and 42 days after injury, and evaluated by independent orthopaedic surgery residents under the direct supervision of at least one experienced orthopaedic surgeon.

### Total RNA extraction from traumatized tissues, quantitative reverse transcription-polymerase chain reaction (qRT-PCR) and common cytokine PCR array

Total RNA from rat (n = 4, 7-days post-injury) and human (n = 5, average 10-days post-injury) blast injured tissues were prepared using the RNeasy mini Kit (Qiagen, Germantown, MD) following manufacturer’s instruction and as previously described [24]. 500 ng of total RNA from each sample was converted into complementary DNA (cDNA) using the Superscript III First Strand Synthesis System for RT-PCR (Invitrogen/Thermo Fisher Scientific, Carlsbad, CA). To investigate common genes related to osteogenesis and fibrosis between human and rat, real-time qRT-PCR was performed using commercially available TaqMan gene expression assays (Applied Biosystems/Thermo Fisher Scientific, Foster City, CA) for *OPN*, *RUNX2* and *COL1A1* (human samples) as well as *Opn*, *Runx2* and *Col1a1* (rat samples). Gene expression was normalized to *HPRT1* (human samples) and *Hprt1* (rat samples) levels, and relative gene expression was determined using the 2^−ΔΔCt^ method [25]. For the cytokine gene expression profiles, RNA integrity was assessed electrophoretically using a 6000 RNA Pico lab-on-a-chip in a Bioanalyzer (Agilent, Santa Clara, CA), and cDNA was synthesized using the RT^2^ First Strand Kit (SA Biosciences/Qiagen). PCR arrays were performed with a RT^2^ Profiler™ PCR Array Rat Common Cytokines (cat# PARN-021Z, SA Biosciences/Qiagen) and RT^2^ Profiler™ PCR Array Human Common Cytokines (cat# PAHS-021Z, SA Biosciences/Qiagen) following manufacturer’s instructions, and data analysis was performed using the RT^2^ Profiler PCR Array Data Analysis software (SA Biosciences/Qiagen). All PCR-based assays were performed using an ABI7900HT system (Applied Biosystems/Thermo Fisher Scientific).

### Histology and picrosirius red (PSR) staining

Both sets of rat and human traumatized tissue and uninjured control tissue samples were separately fixed in phosphate-buffered 4% paraformaldehyde (FD Neuro Technologies Inc, Columbia, MD) followed by sequential ethanol dehydration infiltrated with xylenes and embedded in paraffin as previously described [24]. Five-micron thick sections on glass slides from all tissues were used for hematoxylin and eosin (H&E) and Picrosirius Red (PSR) staining for histo-pathological evaluation and collagen immunohistochemistry, respectively. Sections were deparaffinized in xylenes, rehydrated using a graded series of ethanol and stained with H&E staining following standard laboratory procedures and PSR for 1 h as previously described [24, 26]. Stained H&E slides were analyzed in bright-field microscopy and stained PSR slides were analyzed by polarizing microscopy following standard procedures.

### Scanning electron microscopy (SEM)

Approximately 5 mm^3^ pieces of both traumatized rat and human tissues were decellularized in 1% SDS solution for 30 min at 37 °C. Samples were fixed in 2.5% paraformaldehyde (PFA)/glutaraldehyde in 0.1 M sodium cacodylate buffer (pH 7.4; Electron Microscopy Sciences, Hatfield, PA) overnight at 4 °C. Fixed tissues were incubated with 1% osmium tetroxide (Electron Microscopy Sciences) for 20 min and dehydrated in a graded ethanol (25/50/75/85/95/100%) and hexamethyldisilazane (HMDS; Electron Microscopy Sciences) (25/50/75/100%). Samples were vacuum dried at room temperature. The desiccated samples were coated with gold using a sputter coater (Balzers; Schaumburg, IL) and surface topography was examined by scanning electron microscope (S-4800; Hitachi, Troy, MI, in the Biomedical Engineering and Physical Science Shared Resource, NIBIB, NIH) at 5 kV with various magnifications.

### Immunohistochemistry-immunofluorescence (IHC-IF)

Paraformaldehyde fixed 5-μm thick tissue sections on glass slides underwent deparaffination and hydration and antigen retrieval. Primary antibodies used were Transforming Growth Factor-β1 (TGF-β1) and CD31 (Abcam, Cambridge, MA). After primary antibody incubation, the sections were washed 3 times with phosphate-buffered saline (PBS) for 5 min. The sections were than incubated with each respective secondary antibody (Alexa Fluor 488, Alexa Fluor 568-tagged secondary mouse or rabbit IgG antibodies, Invitrogen/Thermo Fisher Scientific, 1:250) for 30 min at room temperature. After secondary antibody incubation, Hoechst 33342 (1 μg/ml) was applied for 5 min onto section for nuclear staining. The sections were then washed 3 times in PBS for 5 min each and mounted in FluorSave reagent (Calbiochem, Billerica, MA). Slides with stained sections were viewed and analyzed using a Zeiss Axio Observer Z1 with Apotome optical sectioning device (Carl Zeiss, Thornwood, NY).

### Western blot analysis

Rat (n = 4, 7-days post injury) and human (n = 5, average 10-days post-injury from HO patients) traumatized tissues were homogenized in RIPA Buffer (Sigma-Aldrich, St. Louis, MO) containing protease inhibitors (Roche, South San Francisco, CA). Total protein extracts were centrifuged at 13,000 rpm for 15 min at 4 °C, and the supernatants used for downstream analyses. Control uninjured human muscle tissue lysate was purchased from Abcam (Cambridge, MA) and control rat tissue was obtained from the lower uninjured extremities of Sprague–Dawley rats not exposed to blast injury. 10 µg of total protein extracts were separated by gel electrophoresis using a NuPAGE^®^ 4–12% Bis–Tris Gel (Applied Biosystems/Life Technologies, Carlsbad, CA). Proteins were then transferred onto Immobilon-P membranes (Millipore, Burlington, MA) and stained with Ponceu S solution (Sigma-Aldrich) to assess transfer efficiency. Membranes were incubated with the indicated antibodies: Fibronectin (FN), SMAD family member 3 (SMAD3), TGF-β1, Plasminogen Activator Inhibitor 1 (PAI-1) and Glyceraldehyde-3-Phosphate Dehydrogenase (GAPDH) as loading control. Detection was performed by incubation with horseradish peroxidase-conjugated mouse or rabbit secondary antibodies (KPL, Gaithersburg, MD; 1:10,000) followed by Immobilon Western Chemiluminescent HRP Substrate Kit (Millipore).

### Statistical analysis

Replicates are expressed as mean ± SD values and significance was calculated by two-tailed Student’s t-test.

## Results

### Comparisons between rat and human traumatized tissues by radiographs and qRT-PCR

**Fig. 1:**
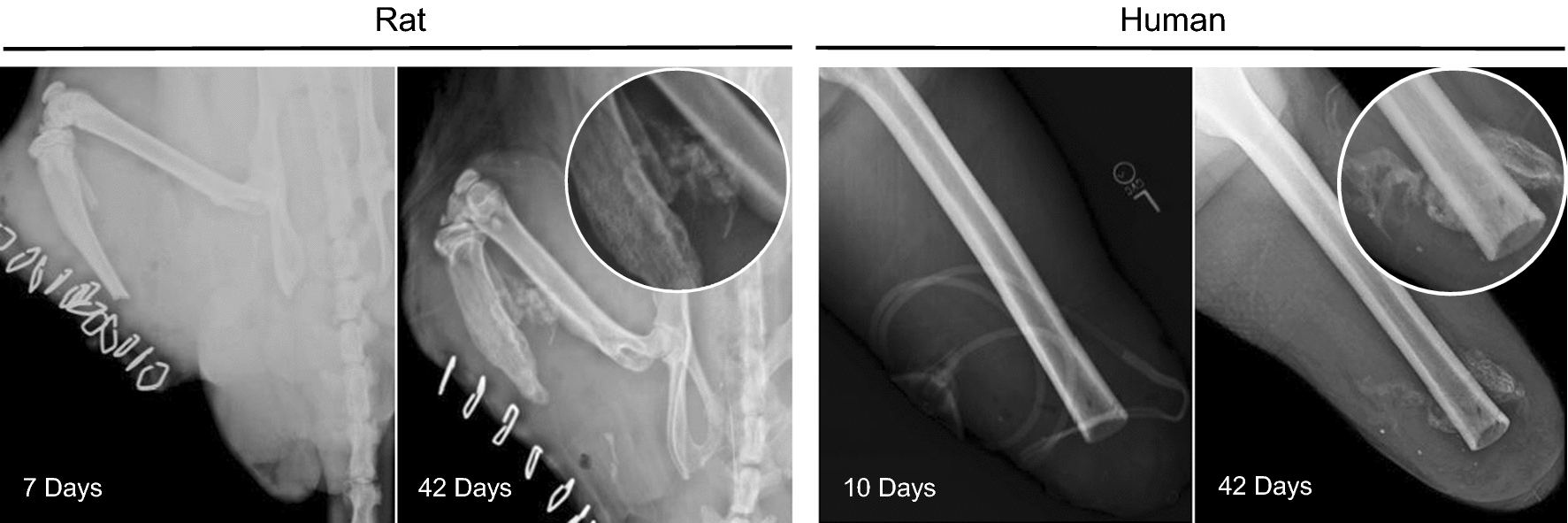
Radiographic images of rat thigh (left) and human femur (right) at 7- or 10- and 42-days post traumatic blast amputation. The insets at 42 days post-injury show regions rich in bone formation within the soft tissue

Recently a rat model has been developed for the formation of traumatic injury-induced HO [16]. Here we sought to examine this model in more detail to determine if morphological, molecular and biochemical similarities existed between traumatized rat and human muscle tissue, specifically focusing on an early fibrotic response. Radiographic analysis demonstrated radio-opaque densities outside the cortical boundaries of the normal skeleton consistent with HO form in both rat (Fig. 1, left panels) and human (Fig. 1, right panels) traumatized tissues. At 7-days post-injury, soft tissue mineralization is evident and these opacities become larger and denser over the 42 days healing period in rat traumatized tissues. Similarly, in human HO, formation of soft tissue mineralization appears at 10-days post-injury and this opacity increases in size and density by 42 days (Fig. 1).

**Fig. 2:**
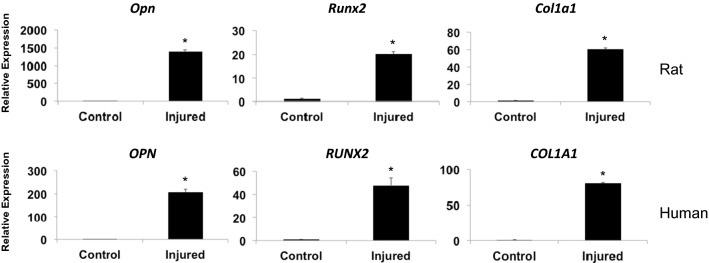
Relative gene expression of the bone related genes *OPN*, *RUNX2* and *COL1A1* as evaluated with q-RT-PCR of RNA from rat muscle tissue at 7-days (upper panels) and human muscle tissue at 10-days (lower panels) following traumatic blast injury. All differences shown are statistically significant (p < 0.01; Student’s *t*-test; error bar = SD)

We next performed qRT-PCR analysis on RNA isolated from HO rat and human tissues to assess the expression of genes associated with early bone formation at 7- to 10-days post-trauma. We detected significant increases (p < 0.01, as evaluated with Student’s *t*-test) in the expression of the osteogenic markers Osteopontin (*Opn* and *OPN*), Runt-related Transcription Factor 2 (*Runx2* and *RUNX2*), and Type 1 Collagen (*Col1a1* and *COL1A1*) in the injured tissues compared to the levels expressed in uninjured control tissues (Fig. 2). This data indicates that the post-blast injured tissues of the rat demonstrate early signs of bone formation nearly identical to that evident in human HO.

### Early histo-pathological comparison between rat and human traumatized tissues demonstrates similar fibrotic development

**Fig. 3:**
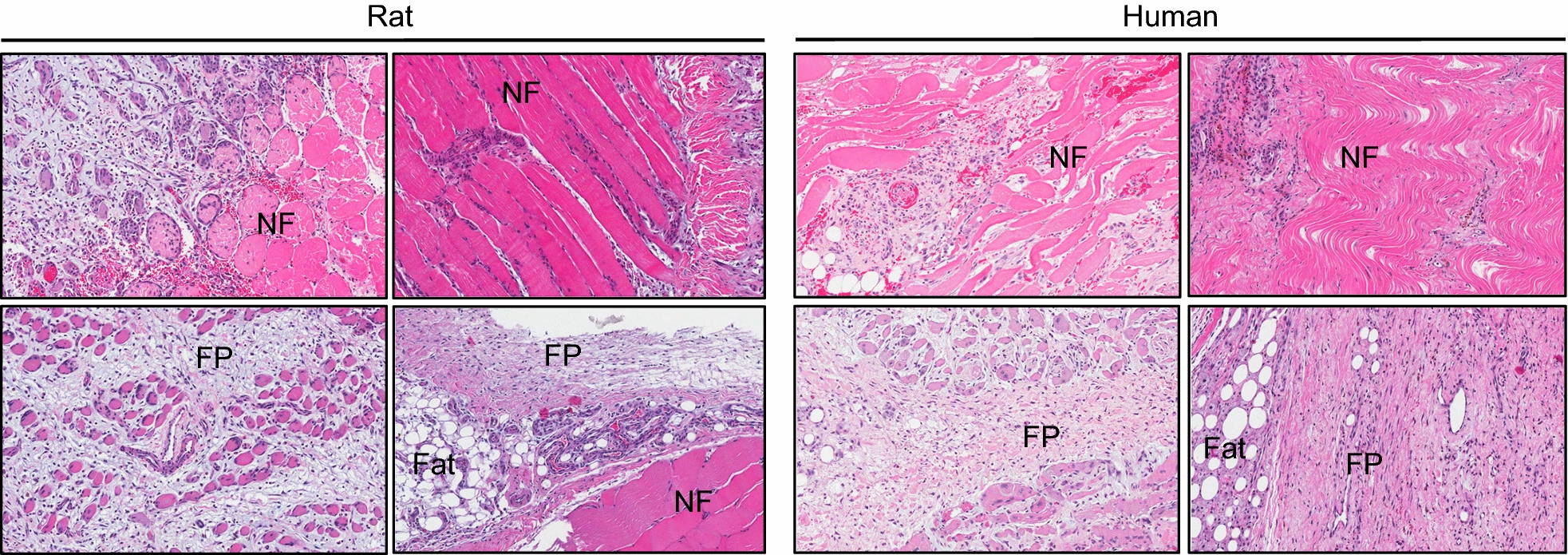
Hematoxylin & eosin stain of rat and human muscle at 7- or 10-days post trauma (NF = normal fibers, FP = fibroproliferative region, Fat = adipose tissue). Stained slides were scanned using Scanscope (Aperio, Vista, California) and images were taken at ×10 magnification

Hematoxylin and Eosin staining revealed that at 7- to 10-days after blast injury, both rat and human tissue exhibited similar patterns of hypercellularity, inflammation and fibrotic hyperplasia (Fig. 3, FP; Fibroproliferative Region, NF; Normal Fiber). Destruction of the normal muscle architecture with fibrous stroma replacing muscle fibers was evident. This hypercellular fibroproliferative response is consistent with that previously reported for the histopathology of human HO tissue samples [24, 27].

**Fig. 4:**
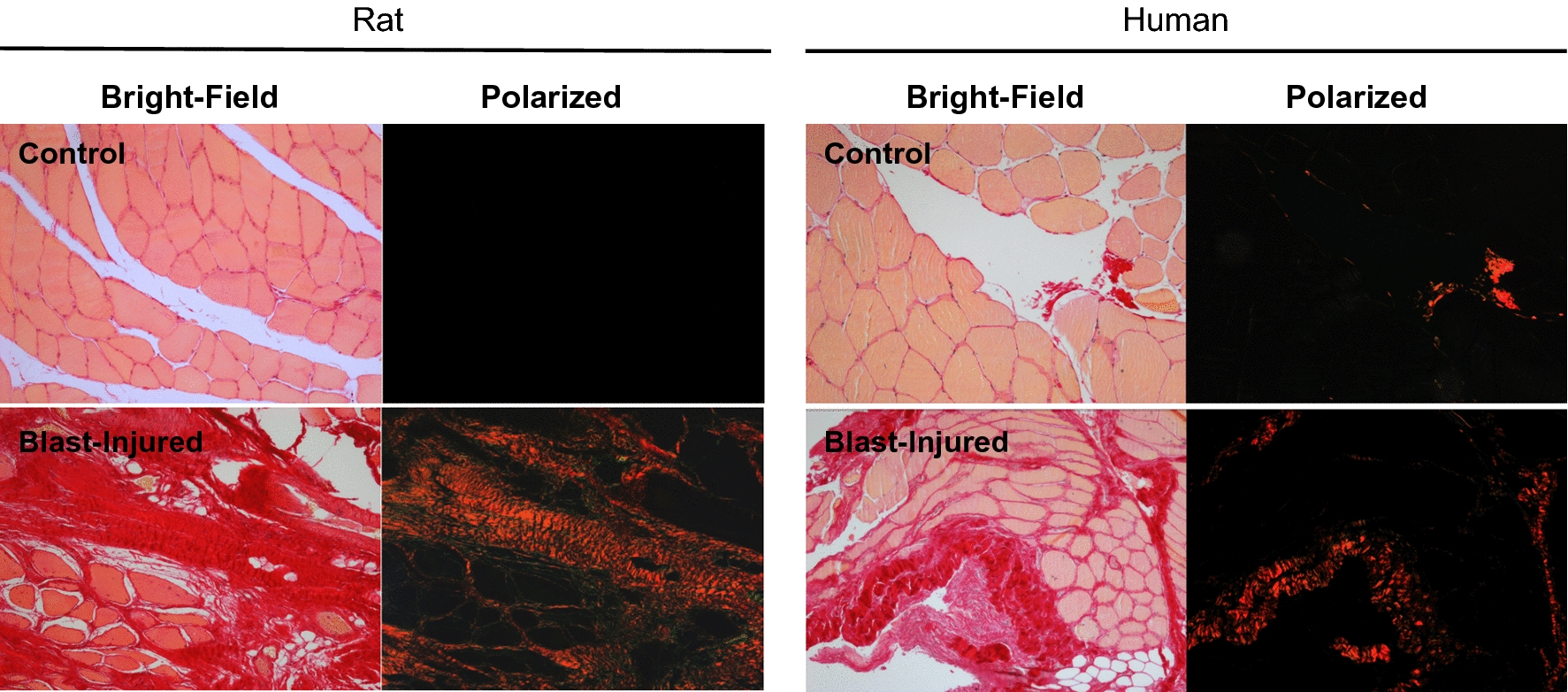
Sections from uninjured control (top panels) and injured (bottom panels) rat and human muscle at 7- or 10-days post trauma. The tissue samples were stained with picrosirius red and photographed under 10X magnification of bright field or polarized light as indicated using Axio scope A1 polarized light microscopy (Carl Zeiss, Thornwood, NY)

We next used picrosirus red (PSR) to stain intramuscular fibrosis. PSR preferentially binds fibrotic collagen fibers (collagens I and III) [26], appearing red under unfiltered light microscopy but also shows enhanced birefringence under polarized light. Both rat and human uninjured control muscle exhibit minimal PSR staining under both bright-field and polarizing conditions (Fig. 4 top panels). However, in the 7- to 10-days post-blast injured tissues there is robust PSR staining within the muscle fibers (Fig. 4, bottom panels), indicating elevated collagen fibril deposition.

**Fig. 5:**
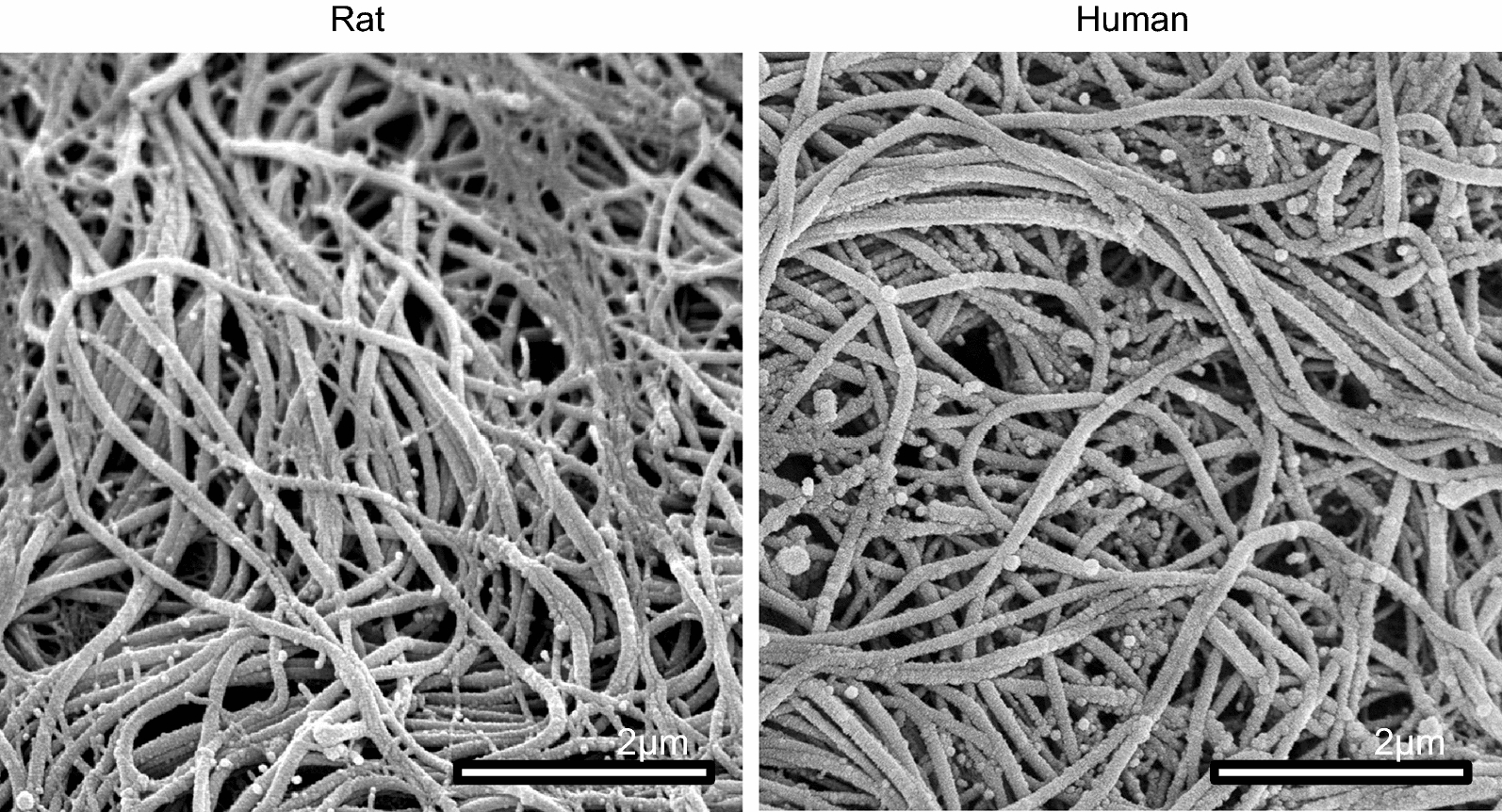
Sections from injured rat (left) and human (right) decellularized muscle at 7- or 10-days post trauma. Scanning electron microscopy was performed on the samples as shown

As an additional method to assess the underlying fibrotic tissue, the injured rat and human muscle was decellularized and processed for scanning electron microscopy. As shown in Fig. 5, the rat and human tissues show a random array of fibers on the order of 100 nm in diameter, consistent with collagen fibrils [28–30], and their arrangement are nearly identical in both samples.

**Fig. 6:**
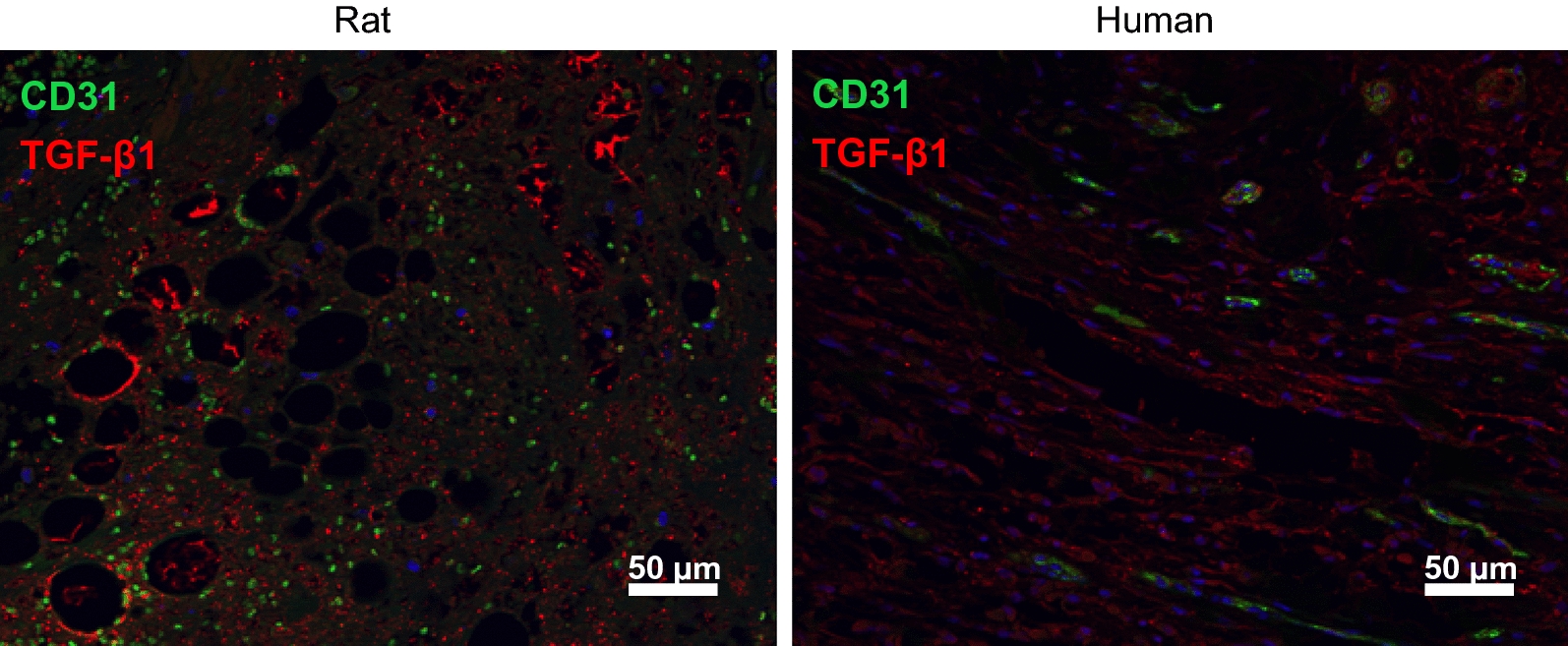
Sections of injured rat (left) and human (right) muscle were processed for immnoflourescent microscopy using antibodies to either TGF-β1 (red) or CD31 (green)

Given that an overt fibrotic response dominates these traumatic wounds, it would be expected that mediators of fibrosis, such as TGF-β family members, would be elevated within the wound area. One such factor, TGF-β1, has been shown to be elevated in HO tissues [24, 31]. We therefore looked at TGF-β1 levels in rat tissues (7-days) and in human tissues (average 10-days) after traumatic injury. As shown in the immunofluorescence in Fig. 6, it is evident that the injured rat and human tissues displayed elevated levels of this pro-fibrotic growth factor. CD31 (PECAM-1), a known marker for endothelial cells that plays a role in angiogenesis during endochondral bone formation [32], was also expressed in both the rat and human samples.

### Comparisons in protein levels and gene expression of target molecules in rat and human traumatized tissues

**Fig. 7:**
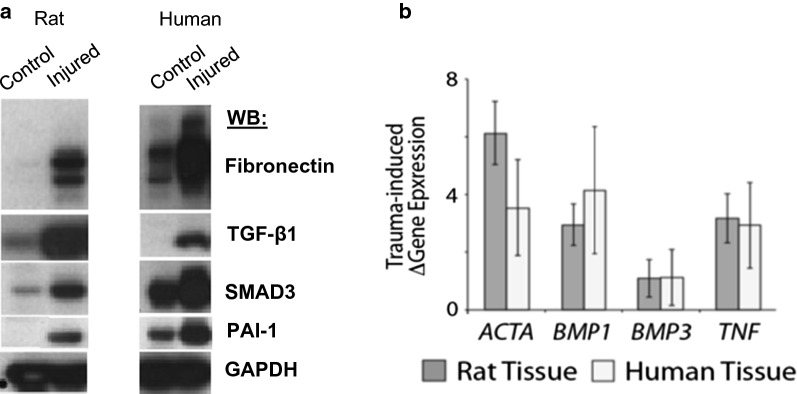
**a** Protein extracts from rat and human uninjured control and blast trauma injured muscle tissue (Rat, 7-days; Human, 10-days HO positive patient sample) were examined by SDS-PAGE and immunoblotting with the indicated antibodies: Fibronectin, TGF-β1, SMAD3 and PAI-1. GAPDH was used as loading control. Presented are data from one representative patient per group. **b** Rat and human tissue demonstrating increase in fold regulation compared to control tissues (error bars = SD). Common cytokine PCR array was performed using RNAs from rat (7-day post-injury) and human (10-day post-injury) muscle tissue following traumatic blast injury

Tissue fibrosis and early bone matrix deposition are common features of HO formation [19–23]. To determine if fibrotic protein markers were present in the injured rat tissue, we analyzed protein samples of injured and uninjured control rat and human tissues by SDS-PAGE followed by immunoblotting. We found significantly elevated expression levels for proteins associated with tissue fibrosis, namely Fibronectin (FN), TGF-β1, SMAD3 and PAI-1 (Fig. 7a). These elevated expression levels were very similar between the rat and human injured tissues.

As an additional way to assess whether the rat and human traumatized tissues showed similarities at the molecular level we screened a cytokine array composed of a number of inflammatory/fibrotic markers with RNA from the traumatized muscle samples. The rat and human tissue samples showed increased expression of *Activin A* (*ACTA*), *Bone Morphogenetic Protein 1* (*BMP1*), *Bone Morphogenetic Protein 3* (*BMP3*) and *Tumor Necrosis Factor α* (*TNF*-*α*), as shown by increased fold regulation compared to control tissue (Fig. 7b). All of these factors have been shown to be involved in the fibrotic response. Overall, our data show remarkable similarity between the rat and human early response after injury in terms of the expression of genes required for tissue fibrosis.

## Discussion

Until recently, there have been few suitable animal models for post-traumatic HO. A key reason for this has been the difficulty in recapitulating the high-energy blast injuries seen too frequently in modern war-conflicts [10–14]. Most previously described animal models of HO are non-physiologic as they induce ectopic bone formation through the use of exogenous agents such as bone morphogenic proteins, bone matrix, or calcium chloride [5, 19, 22], while others induce HO by generating muscle damage though forced manipulation of muscle tissue or Achilles tenotomy [5, 22, 23]. These models produce HO with characteristics similar to that of fibrodysplasia ossificans progressiva (FOP), a genetic disease characterized by a mutation in the type I BMP receptor *ACVR1* [33, 34]. Importantly, while these models are very relevant to investigate the underlying molecular mechanisms of the disease and the discovery of novel targets for therapeutic interventions, they may not reflect the processes following high-energy trauma and do not explain why HO occurs at a uniquely high frequency after combat trauma, particularly given recent evidence that the genes involved in FOP are not up-regulated following these injuries [24]. This study is unique in that we have used a recently developed high-energy blast injury model of HO in rats to directly compare the molecular and cellular events in human patients who have sustained a traumatic blast injury resulting in HO formation. By showing a consistent cascade of early fibrotic and osteogenic events between the rat blast model and post-traumatic human samples that progressed to ectopic bone formation, this study provides a basis for using this animal model in additional basic/mechanistic and pre-clinical studies of post-traumatic HO.

In particular, the utility of this rat model for post-traumatic HO is that it enables one to more rigorously explore the early stages of the disease following the traumatic event. As a result of the inflammation in the wound, endogenous tissue regeneration mechanisms can be overshadowed by a generalized healing response that leads to fibrosis. This early fibroproliferative response has been linked to HO [19–24, 27]. Thus, fibrosis appears to be a salient feature in the HO lesion. Additionally, repeated anecdotal surgical observations have linked areas of abundant fibrotic scarring within the wound to an increased risk of HO formation in humans. Fibrosis may therefore be an intermediate step in the onset of HO in a way that is not completely understood [19–24, 27]. For example, it is possible that regions of fibrosis convert directly to bone or that regions of fibrosis contribute osteogenic signals that drive neighboring, multipotential cells to proceed down a bone-forming pathway.

Our data show that the blast-induced HO in rats, as determined radiographically, occurs through the zone of injury within a similar temporal context as human blast-induced HO. The structural and ultrastructural microenvironment of post-blast tissue visualized by histology is similar in appearance between both rat and human samples. Specifically, collagen fiber orientation and size, muscle tissue destruction and intramuscular fibrosis, are parallel between the rat model and human disease. Additionally, protein analysis of rat and human tissue obtained from the blast injury site demonstrated evidence of fibrosis. This data is consistent with our group’s previous findings of fibrotic precursor elements in patients who develop HO [24, 27, 31, 35]. In particular, Fibronectin, SMAD3, PAI-1 and TGF-β1 demonstrated significantly elevated levels of protein expression in the rat and human blast injured tissue. By screening for markers of inflammatory cytokines that mediate fibrosis, it was found that *ACTA*, *BMP1*, *BMP3* and *TNF*-*α* were elevated at the mRNA level. Importantly all of these markers of fibrosis appear to be important for the aberrant tissue regeneration pathway leading to osteogenesis [24, 27, 31, 35]. This is evident by the elevation in expression of genes needed for osteogenesis at a later time point. We found that *OPN*, *RUNX2* and *COL1A1* were elevated in both rat and human tissue at 7- and average 10-days after blast injury. These data would indicate that the rat blast early response to injury closely parallels that of the human early response to injuries, in terms of markers for fibrosis and osteogenesis in both their temporal and spatial expression.

Blast-induced post-traumatic HO is associated with a variety of systemic biochemical signals that are generated to regulate the hemodynamic, metabolic and immune responses within regions of injury [31]. A likely sequence of events that occurs after injury is that activated progenitor cells within the injured muscle begin to aggregate and proliferate [27]. These cells would then provide trophic and cellular support to the regenerating tissue. A fraction of the cells then receives physical and biochemical cues to become osteoprogenitors and begin to generate ectopic bone.

## Conclusions

The data presented here suggest that the early mechanisms of ectopic bone formation in both rats and humans after initial injury may follow a common pathway via an initial fibroproliferative lesion where osteoinductive, osteoconductive and osteogenic factors are present. By validating these aspects of ectopic bone formation in this trauma-induced rat, we can now more reliably use this model to identify the key events that trigger HO formation, and develop novel biomarkers, such as elevated levels of specific TGF-β family members that can predict HO development in certain patients as well as discover and test novel targets for novel therapeutic interventions, such as inhibitors of the SMAD pathway, to prevent the formation of HO.

## Data Availability

All data generated or analyzed during this study are included in the published article.

## Abbreviations

ACTA: activin A gene
ACVR1: activin A receptor type I
BMP1: bone morphogenetic protein 1 gene
BMP3: bone morphogenetic protein 3 gene
CD31: cluster of differentiation 31, also knows as platelet endothelial cell adhesion molecule (PECAM-1)
cDNA: complementary DNA
COL1A1: type 1 collagen gene
FN: fibronectin
FOP: fibrodysplasia ossificans progressiva
FP: fibroproliferative region
GAPDH: glyceraldehyde-3-phosphate dehydrogenase
H&E: hematoxylin and eosin
HO: heterotopic ossification
HPRT1: hypoxanthine phosphoribosyltransferase 1 gene
IHC-IF: immunohistochemistry-immunofluorescence
mg/kg: milligram/kilogram
μm: micrometer
μg/ml: microgram/milliliter
NF: normal fiber
OPN: osteopontin gene
PAI-1: plasminogen activator inhibitor 1
PBS: phosphate-buffered saline
PCR: polymerase chain reaction
PECAM-1: platelet endothelial cell adhesion molecule, also knows as cluster of differentiation 31 (CD31)
PFA: paraformaldehyde
PSR: picrosirius red
qRT-PCR: quantitative reverse transcription-polymerase chain reaction
RNA: ribonucleic acid
RT: reverse transcription
RUNX2: runt-related transcription factor 2 gene
SDS: sodium dodecyl sulfate
SDS-PAGE: sodium dodecyl sulfate polyacrylamide gel electrophoresis
SEM: scanning electron microscopy
SMAD3: SMAD family member 3
TGF-β1: transforming growth factor-β1
TNFα: tumor necrosis factor α gene
